# Coumarin-Modified CQDs for Biomedical Applications—Two-Step Synthesis and Characterization

**DOI:** 10.3390/ijms21218073

**Published:** 2020-10-29

**Authors:** Łukasz Janus, Julia Radwan-Pragłowska, Marek Piątkowski, Dariusz Bogdał

**Affiliations:** Department of Biotechnology and Physical Chemistry, Faculty of Chemical Engineering and Technology, Cracow University of Technology, Warszawska 24 Street, 31-155 Cracow, Poland; j.radwan@doktorant.pk.edu.pl (J.R.-P.); marek.piatkowski@pk.edu.pl (M.P.); pcbogdal@cyf-kr.edu.pl (D.B.)

**Keywords:** nanomedicine, carbon quantum dots, coumarins

## Abstract

Waste biomass such as lignin constitutes a great raw material for eco-friendly carbon quantum dots (CQDs) synthesis, which find numerous applications in various fields of industry and medicine. Carbon nanodots, due to their unique luminescent properties as well as water-solubility and biocompatibility, are superior to traditional organic dyes. Thus, obtainment of CQDs with advanced properties can contribute to modern diagnosis and cell visualization method development. In this article, a new type of coumarin-modified CQD was obtained via a hybrid, two-step pathway consisting of hydrothermal carbonization and microwave-assisted surface modification with coumarin-3-carboxylic acid and 7-(Diethylamino) coumarin-3-carboxylate. The ready products were characterized over their chemical structure and morphology. The nanomaterials were confirmed to have superior fluorescence characteristics and quantum yield up to 18.40%. They also possessed the ability of biomolecules and ion detection due to the fluorescence quenching phenomena. Their lack of cytotoxicity to L929 mouse fibroblasts was confirmed by XTT assay. Moreover, the CQDs were proven over their applicability in real-time bioimaging. Obtained results clearly demonstrated that proposed surface-modified carbon quantum dots may become a powerful tool applicable in nanomedicine and pharmacy.

## 1. Introduction

Currently, the most efficient and fastest commercially applied diagnostics methods rely on the fluorescence (PL) phenomena of organic compounds due to their superior sensitivity resulting from chemical interactions between analytes and functional groups as well as high fluorescence quantum yield (QY) [1,2]. The application of fluorescence-dependent sensors enables performance of non-invasive biomolecules detection which has multiple advantages such as predictable and easy in interpretation results, fast response, as well reversibility. Such methods are also often associated with long shelf life of the products. However, not all substances are good candidates for biosensors since some of them may exhibit photobleaching or photoblinking in time which is an undesirable feature [3,4,5,6,7]. In addition, they cannot be used for multicolor detection due to the overlapping emission lines [8]. One of the most interesting classes of nanomaterials are quantum dots (QDs) defined as particles of size below 10 nm [3]. Importantly, they exhibit some very attractive features like high fluorescence intensity dependable on the excitation wavelength [3,4,5,6,7]. This unique property is related mostly to various quantum effects [9].

For many years, nanodots with metallic core of semiconductor nature such as zinc sulfide, zinc selenium, cadmium selenium and others where extensively studied over their applicability in photovoltaics [9,10,11], photocatalysis [12,13,14] or photosensing [15,16,17]. They can be also used for bioimaging or biodetection [18,19,20,21,22]. Importantly, aforementioned nanomaterials may exhibit various cytotoxic effects such as reactive oxygen species generation leading to nucleic acids, proteins and mitochondria damage, accumulation on cells surface causing their proper functionality disturbance or metallic ions leakage. They also undergo chemical structure changes under in vivo conditions. Firstly, a metallic core consists of cadmium or selenium which are known to be toxic to both humans and animals even at micromolar concentrations since they inactivate essential mitochondrial proteins [8]. Noteworthy, oxygen accessibility plays an important role since it leads to the oxidation of core atoms. On the other hand, the H^+^ ions may protonate the ligands present at the QDs surface resulting in their cleavage [8,23]. Another obstacle in their biomedical application is their low water solubility as well as poor stability in aqueous media. Thus, there are still many problems to be solved before their successful commercial use in medicine and pharmacy [8].

Current attempts in the increase of quantum dots biosafety rely mostly on their conjugation with synthetic polymers, peptides, lipids or enzymes. The use of poly(ethylene glycol), poly(lactic acid), poly(glycolic acid), poly(vinyl alcohol), poly(methyl methacrylate) enables formation of the functionalized QD core–shell structures with increased hydrophobicity and high potential in nanomedicine applications such as prevention, diagnostics and treatment. Such modifications can be obtained as a result of covalent bond formation, physical adsorption, chelation or some electrostatic interactions. Importantly, a side-effect is a significant increase of the nanodot size which may affects its multiple properties such as the bandgap of the nanocrystals [8].

Recently, carbon quantum dots (CQDs) have gained a lot of scientists’ attention since they are free from the above-mentioned disadvantages while preserving tunable fluorescence of high quantum yield as well as resistance to photobleaching and photoblinking. On the contrary to conventional QDs, the nanodots with a carbon core are water-soluble and non-toxic [24,25]. Moreover, they may undergo numerous modifications by facile methods due to the presence of hydrophilic functional groups on their surface. They can be prepared via two different approaches. The first one known as top-down is slowly withdrawn from use due to the problems with their scalability, expensive equipment and necessity of products post-modification due to the unsatisfactory QY. The second group of CQD preparation methods is called bottom-up and is characterized by facility, low-cost and versatile raw materials which can be used for the synthesis. Until now, CQDs were obtained from many different carbon-rich materials, including waste biomass such as fruit peels, vegetables, coffee grounds or even milk [24]. Thus, they are gradually replacing semiconductor-based nanomaterials in both biomedical, electrooptical and environmental applications including solar cells development [26], in vivo bioimaging [25], metal ions [27] or even microorganisms’ detection [28].

Coumarins constitute a class of polyphenolic naturally occurring organic and synthetic dyes with interesting biological properties such as antimicrobial, anti-inflammatory and antitumor activity as well as antioxidant properties [29,30]. They are characterized with prominent luminescence properties; thus, they have been used as fluorescence probes for bioimaging [31,32]. Currently, they are also used as precursors or components of new compounds or materials for both biomedical and industrial applications [33,34].

In this article, a successful attempt was made to obtain a novel, hybrid carbon quantum dot functionalized with coumarin derivatives using lignin as a carbon source. Chemical structure was confirmed by NMR and FT-IR methods. The obtained nanomaterials had spherical morphology and size below 10 nm typical for CQDs. The CQDs were confirmed to be non-toxic to L929 mouse fibroblasts and capable of the real-time cell visualization under UV light.

## 2. Results and Discussion

### 2.1. Chemical Structure Analysis

**Figure 1:**
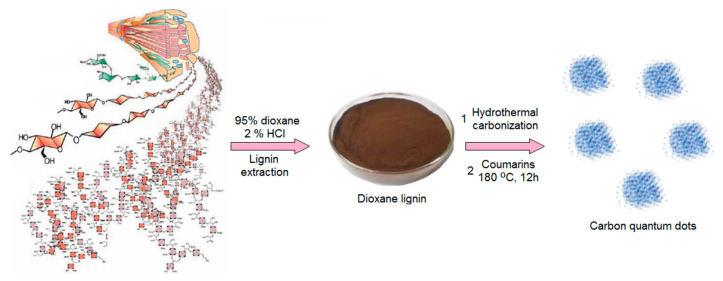
General strategy for carbon quantum dots (CQDs) preparation.

The hybrid nanomaterials for biomedical application were prepared by two-step method. The raw carbon quantum dots were obtained from lignin via hydrothermal synthesis and further modified with two different coumarins under microwave radiation (Figure 1). The general route for CQDs formation consists of three steps namely dehydration, carbonization and passivation. Lignin, a wood-derived polymer contains numerous crosslinked phenylpropane mers. Thus, it is rich in hydroxyl groups. However, without further modification, such as N-doping or grafting, its quantum yield may be insufficient for biomedical applications [35,36]. To enhance fluorescence of the lignin-derived CQDs, the semi-products were subjected to functionalization with two different coumarins.

**Figure 2:**
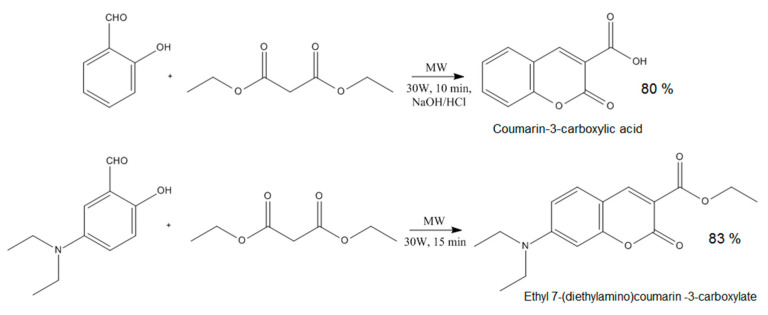
Coumarin derivatives’ synthesis pathway.

To obtain CQDs with enhanced fluorescence quantum yield, two different coumarins were obtained, namely coumarin-3-carboxylic acid and 7-(Diethylamino) coumarin-3-carboxylate which contained free functional groups capable of covalent bonds formation with hydroxyl groups present on the CQDs surface (Figure 2).

**Figure 3:**
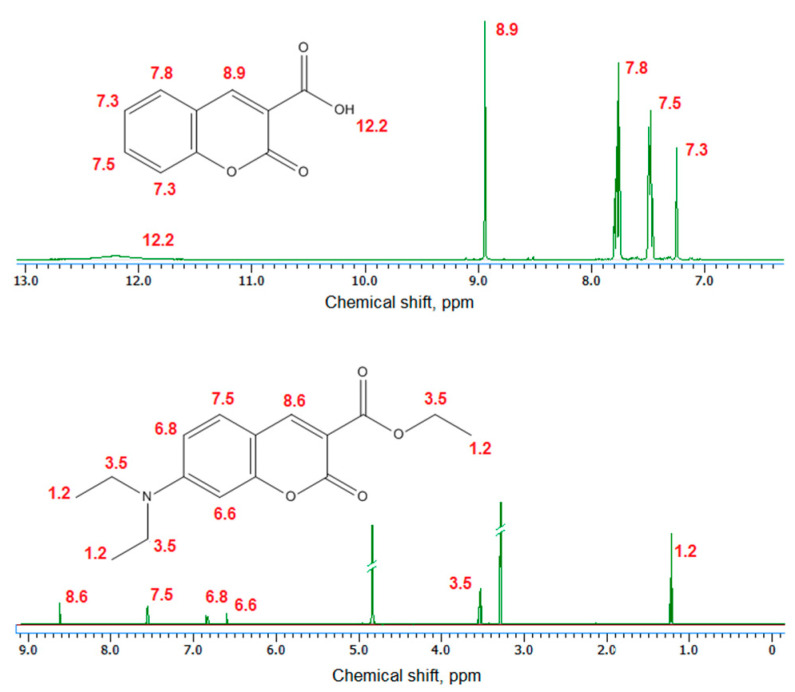
NMR spectra of the obtained coumarin derivatives.

Figure 3 presents ^1^H NMR spectra of the coumarin-3-carboxylic acid and 7-(Diethylamino) coumarin-3-carboxylate. The obtained data for the products correspond to other researchers’ data [37].

**Figure 4:**
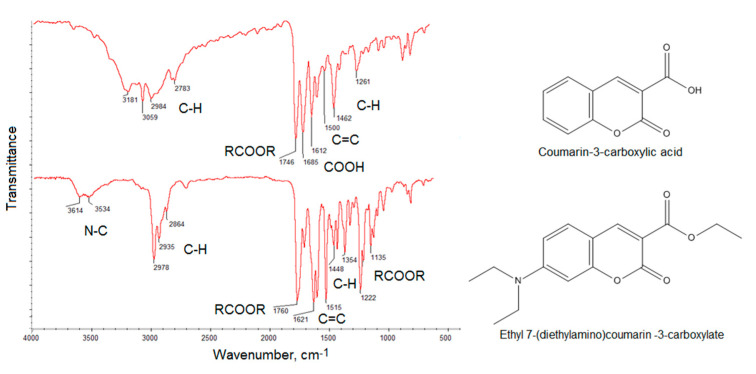
FT-IR spectra of the obtained coumarin derivatives.

Figure 4 presents FT-IR spectra of the coumarin-3-carboxylic acid and 7-(Diethylamino) coumarin-3-carboxylate. The spectra show bands coming from C–H at 2984 cm^−1^, 2783 cm^−1^, 1462 cm^−1^ and 1261 cm^−1^, −COO- at 1746 cm^−1^, COOH at 1685 cm^−1^ and 1612 cm^−1^ and 1500 cm^−1^ coming from unsaturated C = C for the coumarin-3-carboxylic acid. The bands with the maximum at 3181 cm^−1^ and 3059 cm^−1^ also correspond to unsaturated C = C bonds. The FT-IR spectrum of the second compound shows bands typical for N–C at 3614 cm^−1^ and 3534 cm^−1^, C–H at 2978 cm^−1^, 2935 cm^−1^, 2864 cm^−1^, 1448 cm^−1^ and 1354 cm^−1^, -COO- at 1760 cm^−1^, 1222 cm^−1^ and 1135 cm^−1^ as well as C = C at 1621 cm^−1^ and 1515 cm^−1^. The results correspond to those given in the NMR spectra thus confirming aforementioned coumarin derivatives obtainment and their high purity [37].

**Figure 5:**
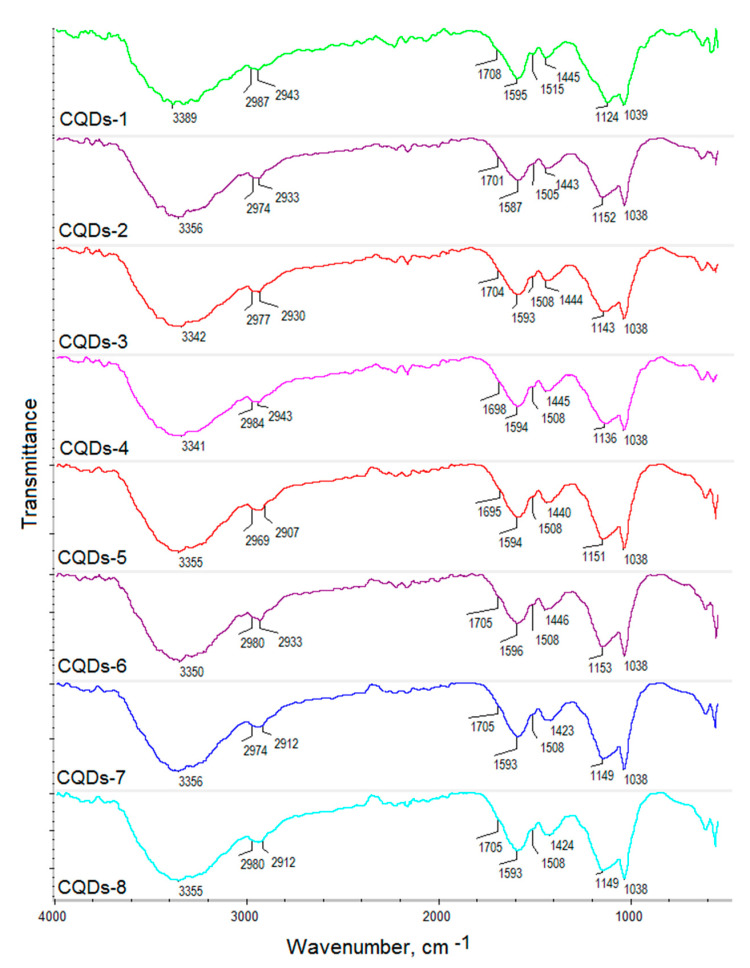
FT-IR spectra of the prepared coumarin-modified CQDs.

The coumarin derivatives described in Figure 2, Figure 3 and Figure 4 were further used for CQDs surface modification as shown in Figure 1. Figure 5 presents the FT-IR spectra of the coumarin-modified carbon quantum dots prepared using lignin as a raw material. The samples were different in terms of the modifying agent and functionalization time. Samples CQDs-1–CQDs-4 were modified with coumarin-3-carboxylic acid for 2, 5, 10 and 20 min, respectively, while samples CQDs-5–CQDs-8 were modified with 7-(Diethylamino) coumarin-3-carboxylate for 2, 5, 10 and 20 min, respectively. The FT-IR spectra are typical for CQDs [35,36,38,39]. All of the samples show the broad bands coming from free carboxylic and hydroxylic groups ranging from 3389 cm^−1^ to 3341 cm^−1^ responsible for hydrophilic properties of the CQDs. There are bands typical for -CH_2_- and -CH_3_ groups visible from 2987 cm^−1^ to 2969 cm^−1^ and from 2943 cm^−1^ to 2907 cm^−1^ as well as aromatic ring -CH and C=O typical for lignin structure from 1596 cm^−1^ to 1593 cm^−1^, from 1423 cm^−1^ to 1446 cm^−1^, from 1124 cm^−1^ to 1153 cm^−1^, as well as from 1039 cm^−1^ to 1038 cm^−1^ [39]. The successful modification of the nanodots with coumarin derivatives confirms the formation of ester bonds between carboxylic groups coming from coumarin-3-carboxylic acid and 7-(Diethylamino) coumarin-3-carboxylate and free hydroxyl groups coming from CQDs surface. The incorporation of coumarins also confirms the presence of the bands coming from C=C (1515 cm^−1^–1508 cm^−1^) and aromatic rings [37].

### 2.2. Morphology Analysis

**Figure 6:**
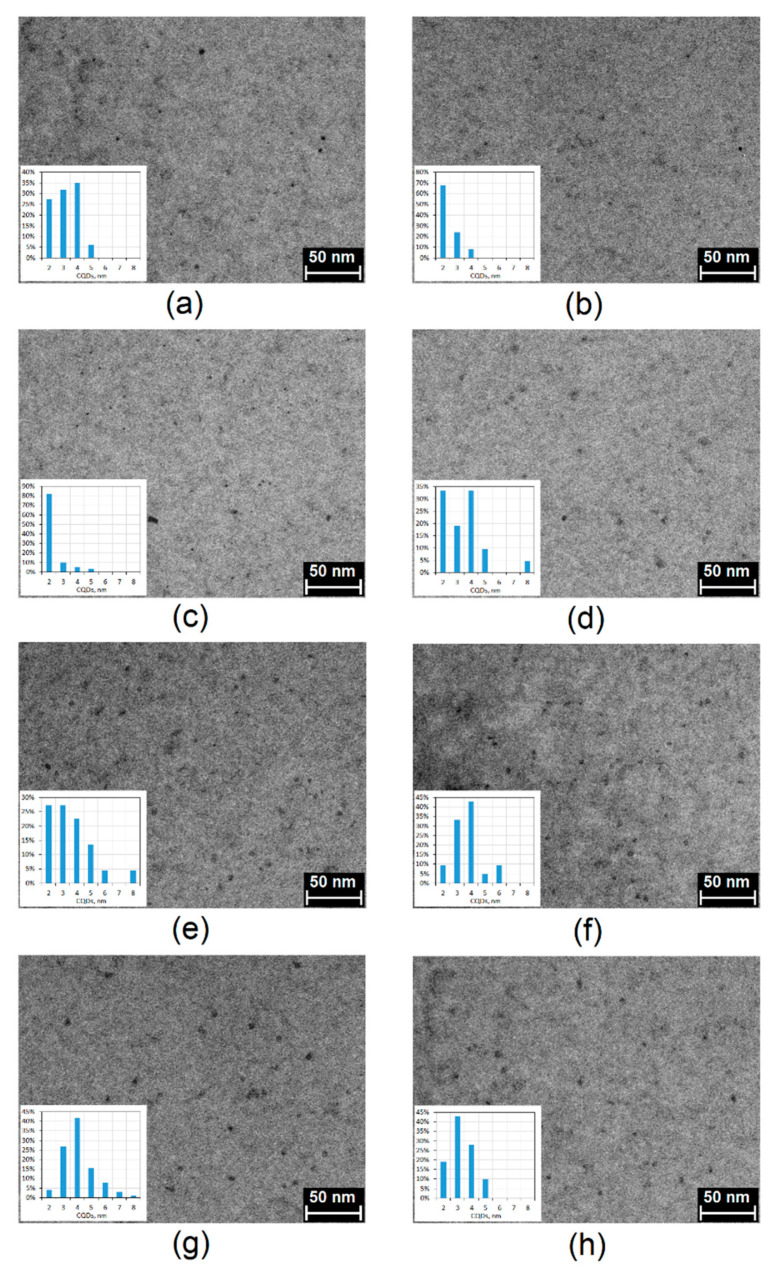
TEM images of the prepared CQDs: (**a**) CQDs-1; (**b**) CQDs-2; (**c**) CQDs-3; (**d**) CQDs-4; (**e**) CQDs-5; (**f**) CQDs-6; (**g**) CQDs-7; (**h**) CQDs-8.

The morphology analysis of the prepared CQDs was investigated by transmission electron microscopy (TEM) technique. As shown in Figure 6, in all cases carbon quantum dots were obtained at a size below 10 nm and were well dispersed in water. It may be observed that the nanodots are uniformly dispersed ad of quite low size polydispersity and have lattice fringes characteristic for CQDs [35,36]. Their average size is 2–5 nm. They have spherical shape typical for carbon nanomaterials prepared from waste biomass [24,35,36].

### 2.3. Optical Properties Analysis

**Figure 7:**
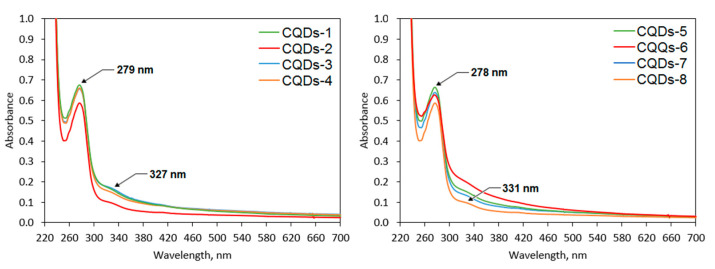
UV–Vis spectra of the coumarin-modified CQDs.

Figure 7 presents the UV–Vis spectra of the prepared nanomaterials. Appropriate optical parameters are crucial for their future biomedical applications. It can be noticed that the CQDs exhibit a well-defined band with the maximum peaks which corresponds to the aromatic groups (π systems) as well as n→π* transitions typical for carbonyl groups at 327 nm (samples modified with coumarin-3-carboxylic acid) and 331 nm (samples modified with 7-(diethylaminocoumarin)-3-carboxylate)) and π→π* electron transitions of the C=C at 279 nm (samples modified with coumarin-3-carboxylic acid) and 278 nm (samples modified with 7-(diethylaminocoumarin)-3-carboxylate) [35,36]. The bands can be attributed to the polymeric fragments (lignin) of the CQDs as well as to the coumarin-derivatives.

**Figure 8:**
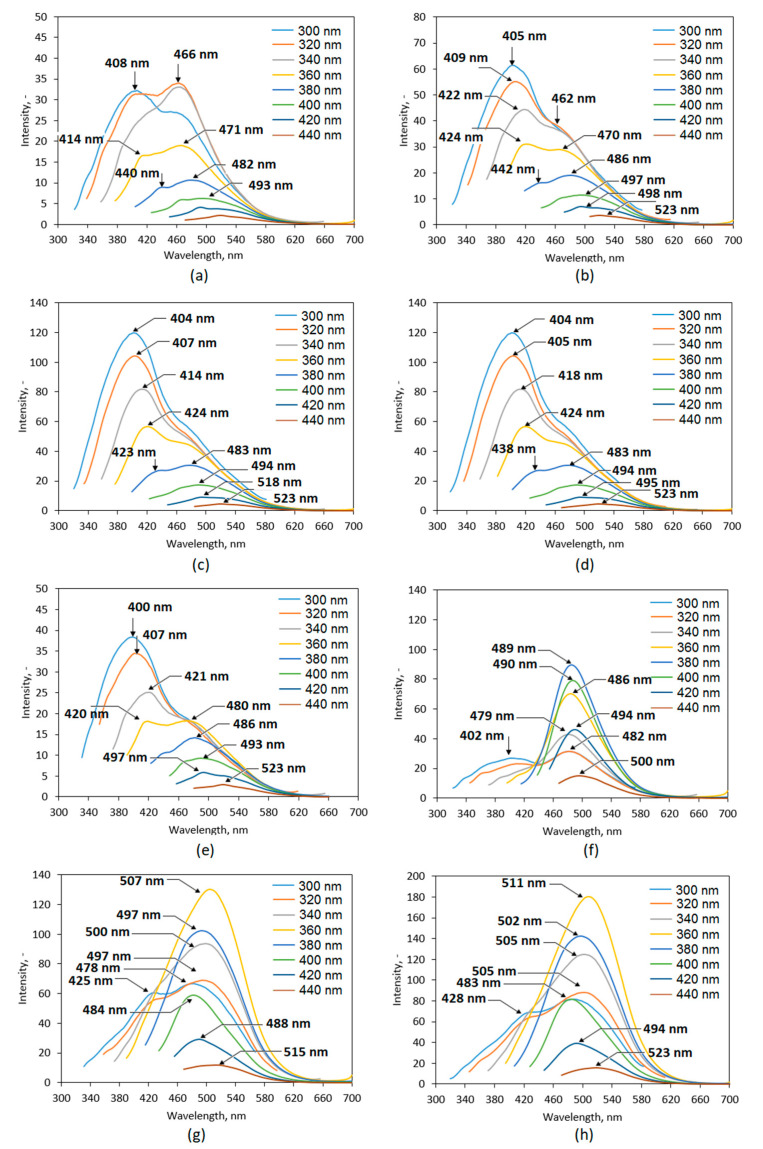
Fluorescence spectra of the prepared CQDs: (**a**) CQDs-1; (**b**) CQDs-2; (**c**) CQDs-3; (**d**) CQDs-4; (**e**) CQDs-5; (**f**) CQDs-6; (**g**) CQDs-7; (**h**) CQDs-8.

The obtained nanomaterials were studied over their fluorescence properties (Figure 8). It is well-known that all carbon dots potentially exhibit very good or excellent luminescent behaviors which depend on the QD type [3,4,5,6,7,8,9,10,11]. PL spectra of the prepared CQDs show that all samples are promising candidates for biomedical optical-based applications. It may be observed that the fluorescence intensity is different between the samples and depends on the reaction parameters. One may observe that PL characteristics depends on both reaction duration as well as the type of coumarin used for modification. Each case the increase in reaction time affects positively PL intensity. Clearly, the best PL properties were obtained for the samples functionalized with 7-(diethylaminocoumarin)-3-carboxylate which can be explained by the presence of triethylamine group in its structure. Importantly, the coumarin-derivatives functionalization resulted in the shift of the peak maximum to the longer wavelengths which is a desired feature for biomedical applications [35]. This effect is visible mostly for the samples modified with coumarin ester for which maximum peak is observed for 507 nm (CQDs-7) and 511 nm (CQDs-8). The most prominent PL is observed for the sample CQDs-8 and is almost five times higher comparing to the sample CQDs-1 and CQDs-5. It may be observed that the surface modification via coumarin grafting has a major effect on PL characteristics. For the samples, the PL emission dependence on the excitation wavelength is observed which a unique feature of QDs [3]. Carbon quantum dots due to the more complicated structure comparing to the nanomaterials with metallic core such as ZnSe or CdSe QDs, thus their PL mechanisms are not yet fully understood. It is assumed that fluorescence of the carbon nanomaterials is caused by three major independent factors, namely crystalline structure disturbance, quantum confinement and surface traps, as well as molecular effect resulting from the presence of aromatic structures or conjugated double bonds sequences. Tunable fluorescence is a highly desired feature for the modern bioimaging and biodetection methods since they provide possibility of a few cellular components’ simultaneous visualization. Presented results suggest that proposed coumarin-modified CQDs may find utility in various biomedical applications [3,7,24,35,36].

The samples were also investigated over their quantum yield which is highly important for applications such as biodetection and bioimaging. Table 1 shows the QY for the obtained carbon quantum dots. The reference was quinine sulfate solution. One may observe, that the results correspond to the PL spectra. The quantum yield is correlated with CQDs post-synthesis modification duration. The QY value is significantly higher for the samples prepared using coumarin ester (up to 18.4%) which is significantly higher compared to CQDs without functionalization [3] and is comparable to QY of the CQDs prepared using toxic reagents such as sodium azide or diethylamine [24,35,36]. All of the nanodots exhibited great photostability in time over 7 and 30 days which clearly confirms their utility in real-time cell bioimaging and biosensing.

**Figure 9:**
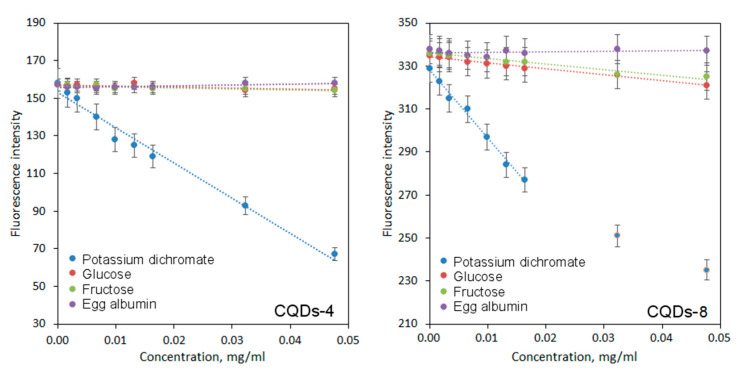
Sensing abilities of coumarin-modified CQDs.

One of the most interesting potential applications of CQDs is biosensing of various ions and substances [35]. Figure 9 presents the results of biodetection study on sugars such as glucose, fructose, model protein–albumin and chromium ions. It may be observed that CQDs exhibit different sensing ability depending on the analite being tested and exhibit selectivity. The regression equations are given in Table 2.

In the case of both glucose and fructose, a decline in fluorescence intensity is observed. On the other hand, no significant changes in PL intensity occur for the albumin. The difference between fluorescence quenching can be explained by the complexity of analytes structure. Both glucose and fructose molecules can approach CQDs more easily and interact with the bigger number of functional groups leading to PL decay. The sensitivity was higher for the CQDs modified with 7-(diethylaminocoumarin)-3-carboxylate which is caused by the possibility of amine group protonation [3]. A strong fluorescence decline occurred for chromium ions, possibly due to charge transfer. Very good sensing properties in this case can be explained by electrostatic interactions between hydroxyl and carbonyl groups of CQDs and Cr^6+^ ions as well as aggregates formation. Another crucial factor could be chelating abilities of CQDs. Again, in this case, the sensitivity was higher for the CQDs modified with 7-(diethylaminocoumarin)-3-carboxylate. The results correspond to other researchers’ data [35].

**Figure 10:**
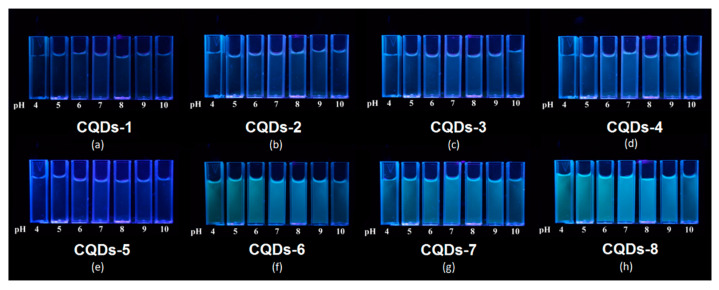
Images of the prepared CQDs’ fluorescence dependence on solvent pH value (excitation wavelength = 365 nm): (**a**) CQDs-1; (**b**) CQDs-2; (**c**) CQDs-3; (**d**) CQDs-4; (**e**) CQDs-5; (**f**) CQDs-6; (**g**) CQDs-7; (**h**) CQDs-8.

Figure 10 presents the PL behavior depending on the solvent pH. The results clearly show that sample CQDs-6, CQDs-7 and CQDs-8 exhibit strong correlation between fluorescence and pH. Sample CQDs-3 and CQDs-4 exhibit moderate sensitivity to pH changes, while samples CQDs-1, CQDs-2 and CQDs-5 show minor or no effect. It can be explained by the differences in chemical structure of the prepared samples, especially presence of hydroxyl and carboxyl groups. Noteworthy, most of the biological fluids’ pH oscillates around 7. The PL intensity change due to the acidity increase of the environment can be helpful especially during cancer cells labeling [36].

### 2.4. In Vitro Cytotoxicity Study

**Figure 11:**
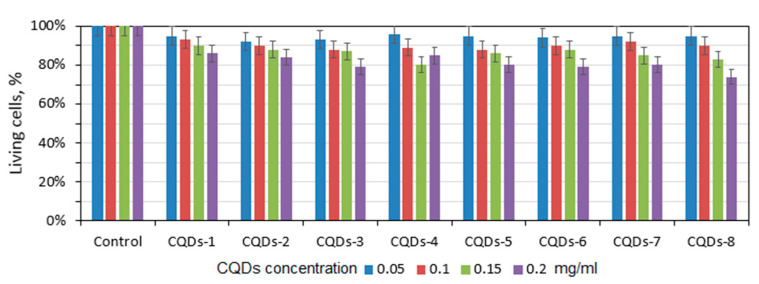
Results of the XTT assay carried out on L929 mouse fibroblasts at four different concentrations: 0.05 mg/mL, 0.1 mg/mL, 0.15 mg/mL and 0.2 mg/mL.

Nanodots which consist of a carbon core are characterized by low or lack of cytotoxicity. Therefore, they find multiple applications in biomedicine and pharmacy. Due to their excellent resistance to photobleaching and photodegradation, they are found to be superior to traditional organic dyes. In the most cases, CQDs undergo functionalization to increase its quantum yield, nevertheless introduction additional molecules or atoms may somehow negatively affect biocompatibility of the nanomaterials. Figure 11 presents results of the XTT assay performed on L929 mouse fibroblasts which are commonly used for in vitro cytotoxicity testing to verify their utility in such applications as cell labeling, bioimaging or biosensing. For the study, four different concentrations were used, namely 0.05–2.0 mg/mL. There is no significant decrease in the cells’ viability even at higher CQDs concentrations which proves that carbon dots superficial modification via coumarin derivatives grafting did not negatively affect its biological properties. Thus, it may be stated that all prepared CQDs exhibit good biocompatibility. Such results correspond to data of other researchers [35,36].

### 2.5. Cells Visualization Study

**Figure 12:**
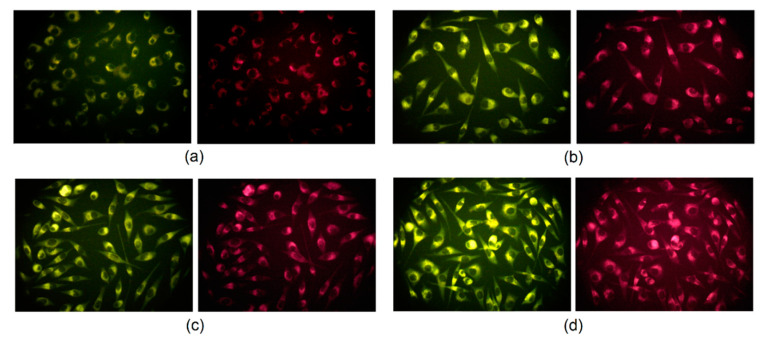
L929 mouse fibroblast visualization after 24 h of incubation: (**a**) CQDs-4 (emission at 520 nm); (**b**) CQDs-6 (emission at 590 nm); (**c**) CQDs-7 (emission at 520 nm); (**d**) CQDs-8 (emission at 590 nm).

Carbon quantum dots, due to their excitation-dependent fluorescence emission, can be used in cell labeling and bioimaging as multicolor fluorescent nanoprobes which is out of reach for conventional organic dyes. Generally, it is known that carbon quantum dots can transfer into the eukaryotic cell via clathrin-mediated endocytosis as well as macropinocytosis. Figure 12 presents the results of the L929 mouse fibroblast visualization by the means of fluorescence microscopy. It may be noticed that CQDs were able to penetrate cell membranes and emitted fluorescence at excitation at 460–490 nm and emission at 520 nm and excitation at 510–550 nm and emission at 590 nm. Noteworthy, the nanodots are present in the cytoplasm whereas there are no CQDs visible inside of the nuclei. It can be explained by the potential interactions between CQD functional groups and proteins forming cytoskeleton such as vimentin [3,35,36].

## 3. Materials and Methods

### 3.1. Materials

Propylene carbonate, dioxane, sulfuric acid, hydrochloric acid, sodium hydroxide, salicylic aldehyde, diethylmalonate, piperidine, *N, N*-diethyl salicylic aldehyde, ethanol, glucose, fructose, sodium citrate, sodium acetate, ammonium chloride, ammonia, potassium dichromate, deuterated methanol were purchased from Sigma Aldrich (Poznań, Polska). Albumin was obtained from egg white. Lignin was derived from dried miscant plant via extraction. XTT (2,3-bis-(2-methoxy-4-nitro-5-sulfophenyl)-2H-tetrazolium-5-carboxanilide) assay, DMEM without phenol red with high glucose, penicillin/streptomycin, trypsin, phosphate buffer, L929 mouse fibroblasts were purchased from Sigma Aldrich (Poznań, Polska). Sterile filters 0.22 µm were purchased from Bionovo (Legnica Poland) and regenerated cellulose membranes for dialysis (MWCO = 1000 Da) were obtained from VWR International (Gdańsk, Poland). Multiwhole plates (96 wholes) were purchased from Genoplast, Rokocin, Poland.

### 3.2. Methods

#### 3.2.1. CQDs Preparation

Carbon quantum dots were obtained using lignin as a raw material, propylene carbonate as a high boiling solvent and sulfuric acid as a carbonizing agent in hydrothermal autoclaves at 180 °C (Table 3). For the coumarin-derivative (coumarin-3-carboxylic acid) obtainment, 12.2 g of salicylic aldehyde, 17.68 g diethylmalonate and 0.2 mL of piperidine (catalyst) were used. The solution was mixed using magnetic stirrer (Chemland) and placed in microwave reactor (Prolabo Synthwave) for 25 min, at 100 °C (power = 45 W). The semi-product (ethyl coumarin-3-carboxylate) was cooled down and purified via double-crystallization using ethanol as a solvent. The last step was ester hydrolysis to obtain acid which was carried out in 60% methanol solution with 10% excess of NaOH to coumarin ester groups (40 °C, 1 h). The product was acidified with HCl to pH = 2, crystalized using pure methanol and dried. To obtain 7-(diethylaminocoumarin)-3-carboxylate, 5 g of N, N-diethyl salicylic aldehyde was mixed with 4.58 g diethylmalonate and 0.5 mL piperidine (catalyst). The reaction was carried out for 6 min at 98 °C (power = 60 W). The product was double-crystalized using methanol. The post-reaction modification of lignin-based carbon quantum dots was carried out under microwave-assisted conditions using Prolabo Synthwave reactor (parameters given in Table 3). The carbon dots were purified using sterile filters (0.22 µm) and dialysis tubing (MWCO = 1000 Da) for 3 days (the water was changed daily).

#### 3.2.2. Chemical Structure Analysis

Coumarin derivatives were analyzed by ^1^H Nuclear Magnetic Resonance (NMR) method (16 scans) using Jeol FP-NMR 500 MHz (Japan). The samples (50 mg) were dissolved using D-methanol and Fourier Transform Infrared Spectroscopy (FT-IR) with ATR adapter (ThermoNicolet, Nexus 470 FT-IR, USA).

#### 3.2.3. Optical Properties Study

The UV–Vis spectra were collected using Agilent 8453 Diode array spectrophotometer [3], whereas fluorescence spectra were collected with the use of Jasco FP-750 spectrofluorometer. The fluorescence quantum yield (*QY*) was determined according to the previously described method using quinine sulfate solution as in reference [3] according to Equation (1):

(1) $$QY_{s}\;=\;Q_{r}\left(\frac{A_{r}}{A_{s}}\right)\left(\frac{E_{s}}{E_{r}}\right){\left(\frac{\eta_{s}}{\eta_{r}}\right)}^{2},$$

*QY* = fluorescence quantum yield;

*ŋ* = refractive index of the solvent;

*A* = absorbance of the solution;

*E* = integrated fluorescence intensity of the emitted light.

Subscripts ‘*r*’ and ‘*s*’ refer to the quinine sulfate (reference) and sample.

Solutions’ pH was determined with Elmetron CX-551 pH-meter. The pH-sensitivity tests were carried out in the range between 4 and 10.

Photostability was determined by placing CQDs solutions in quartz cuvettes which were exposed to the continuous irradiation with the mercury lamp (λ = 365 nm, power = 20 W). The QY was calculated after 7 and 30 days using Equation (1). The sensing ability studies were carried out using CQDs solutions with concentration of 0.20 mg/mL.

#### 3.2.4. Morphology Study

The morphology was studied using Transmission Electron Microscopy (TEM) using JEOL JEM2100 HT CRYO LaB6 microscope. The samples were placed in the form of the aquatic solutions on copper mesh covered with formvar and left to evaporate.

#### 3.2.5. In Vitro Cytotoxicity Study

To determine cytotoxicity of the samples, commercial L929 mouse fibroblasts cell line was applied which is recommended by the ISO 10,993 norm. The cytotoxicity was evaluated using quantitative method—XTT assay after 3 days of cell culture under standard conditions (5% CO_2_, 98% humidity, 37 °C) according to the producer protocol at four different concentrations 0.05 mg/mL, 0.1 mg/mL, 0.15 mg/mL and 0.2 mg/mL using 96-whole plates.

#### 3.2.6. Cells Visualization Study

For real-time cell imaging, the L929 cell was used. For this reason, the cells were incubated with CQDs solutions for 24 h under standard conditions and observed under inverted microscope Delta Optical equipped with fluorescence adapter. The cells were studied using two different filters with excitation at 460–490 nm and emission 520 nm (green fluorescence) as well as excitation 510–550 nm and 590 emission (red fluorescence).

## 4. Conclusions

In this article, a novel type of carbon quantum dots is described. The lignin-based nanodots were successfully modified with the coumarin-3-carboxylic acid and 7-(Diethylamino) coumarin-3-carboxylate under microwave-assisted conditions. The functionalized CQDs exhibited strong fluorescence and high resistance to photobleaching in time. Noteworthy, their PL emission was shifted to the longer wavelengths. The highest quantum yield was obtained for the samples modified with coumarin ester. The nanodots were pH-sensitive and were capable of various molecules as well as chromium ions sensing. Coumarin-modified CQDs were not cytotoxic to L929 mouse fibroblasts and were capable of cell membrane penetration. The nanodots were proven to stain the cytoplasm. Overall, the results clearly demonstrated the proposed novel nanomaterials’ versatility and multiple possible applications in medicine and pharmacy.

## Figures and Tables

**Table 1:** Coumarin-modified CQD quantum yield and its stability in time (7 and 30 days).

Sample	Fluorescence Quantum Yield, %	Photostability after 7 Days, %	Photostability after 30 Days, %
CQDs-1	3.4	3.4	3.3
CQDs-2	6.7	6.6	6.4
CQDs-3	11.2	11.1	11.0
CQDs-4	11.1	11.0	10.6
CQDs-5	3.5	3.3	3.3
CQDs-6	9.8	9.6	9.5
CQDs-7	14.7	14.5	14.2
CQDs-8	18.4	18.1	17.8

**Table 2:** Coumarin-modified CQD sensing sensitivity.

Sample	Analite	Regression Equation
CQDs-4	Potassium dichromate	y = −1874.4x + 153.16
	Glucose	y = −48.505x + 156.82
	Fructose	y = −58.180x + 156.62
	Egg albumin	y = 47.451x + 155.75
CQDs-8	Potassium dichromate	y = −3190.7x + 328.27
	Glucose	y = −279.9x + 334.29
	Fructose	y = −257.02x + 336.07
	Egg albumin	y = 21.161x + 336.14

**Table 3:** Synthesis parameters of coumarin-modified CQDs.

Sample	Lignin, g; Propylene Carbonate, mL; H_2_SO_4_, g	Hydrothermal Carbonization Time, h	Coumarin, g	Reaction Time, min (MW 30 W)
CQDs-1	0.05; 3; 0.05	12	Coumarin-3-carboxylic acid, 0.05	2
CQDs-2				5
CQDs-3				10
CQDs-4				20
CQDs-5			7-(diethylaminocoumarin)-3-carboxylate, 0.05	2
CQDs-6				5
CQDs-7				10
CQDs-8				20
